# Postangioplasty Restenosis Followed with Magnetic Resonance Imaging in an Atherosclerotic Rabbit Model

**DOI:** 10.1155/2012/747264

**Published:** 2012-12-19

**Authors:** Mari Hänni, Olli Leppänen, Örjan Smedby

**Affiliations:** ^1^Department of Radiology, Oncology, and Radiation Science, Section of Radiology, Uppsala University, 751 85 Uppsala, Sweden; ^2^Department of Surgery, Uppsala University Hospital, 751 85 Uppsala, Sweden; ^3^Division of Radiological Sciences (IMH), Linköping University, Linköping, Sweden; ^4^Center for Medical Image Science and Visualization (CMIV), Linköping University, 581 85 Linköping, Sweden

## Abstract

*Rationale and Objectives*. Testing a quantitative, noninvasive method to assess postangioplasty vessel wall changes in an animal model. *Material and Methods*. Six New Zealand white rabbits were subjected to atherosclerotic injury, including cholesterol-enriched diet, deendothelialization, and percutaneous transluminal angioplasty (PTA) in the distal part of abdominal aorta (four weeks after deendothelialization). The animals were examined with a 1.5T MRI scanner at three times as follows: baseline (six weeks after diet start and two days after PTA) and four weeks and 10 weeks after-PTA. Inflow angiosequence (M2DI) and proton-density-weighted sequence (PDW) were performed to examine the aorta with axial slices. To identify the inner and outer vessel wall boundaries, a dynamic contour algorithm (Gradient Vector Flow Snakes) was applied to the images, followed by calculation of the vessel wall dimensions. The results were compared with histopathological analysis. *Results*. The wall thickness in the lesion was significantly higher than in the control region at 4 and 10 weeks, reflecting induction of experimentally created after-angioplasty lesion. At baseline, no significant difference between the two regions was present. *Conclusions*. It is possible to follow the development of vessel wall changes after-PTA with MRI in this rabbit model.

## 1. Introduction

A common way to treat arterial stenoses due to atherosclerosis is percutaneous transluminal angioplasty (PTA), intended to improve the hemodynamic conditions. This therapeutic procedure itself involves an injury to the vessel wall and the repair of the injury starts immediately post-PTA. Sometimes the repair process causes a lumen narrowing that affects the flow unfavourably. To evaluate medication aimed at diminishing the vessel wall reactions there is a need for quantitative, non-invasive methods to follow the repair process in the vessel wall post-PTA. The methods should preferably be applicable to animal models. 

Magnetic resonance imaging (MRI) is a method that is, in general, non-invasive and relatively independent of the operator. We have previously proposed a quantitative method based on a combination of MRI and image processing techniques (thresholding and 3D morphology), that was tested for examining severely atherosclerotic animals [1]. We have also proposed a modified method combining MRI with an alternative image processing technique, the Gradient Vector Flow snake algorithm [2]. This image processing procedure is less operator-dependent and less time-consuming than thresholding and 3D morphology. The results from the combined MRI-snakes method were promising when animals with moderate atherosclerosis were examined [2]. This method may be an option for longitudinal studies in animal models.

The aim of this study was to apply the MRI-snakes method in a rabbit model in order to quantify vessel wall changes after angioplasty. 

## 2. Material and Methods

### 2.1. Animal Model

**Figure 1:**
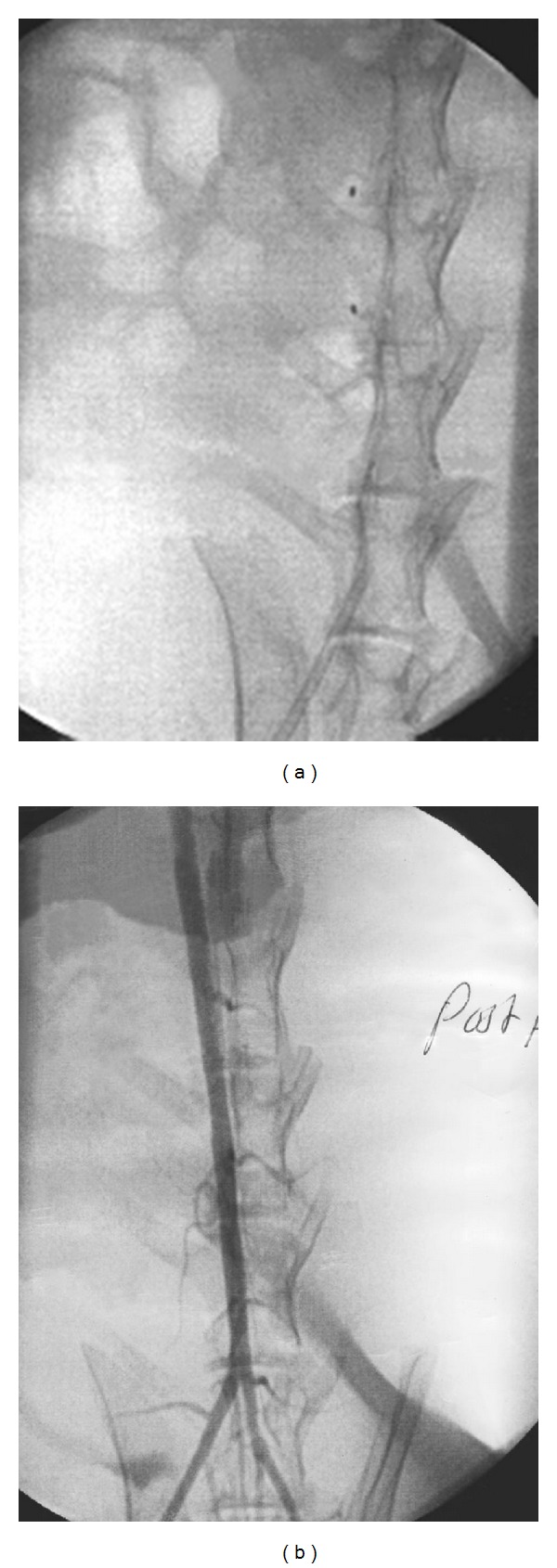
Angioplasty of abdominal aorta. (a) The balloon location is assessed by the markers in the balloon. (b) Post-PTA-angiography, different rabbit than in (a), showing the aortic bifurcation.

A double-injury, cholesterol-fed New Zealand white rabbit model (3.1–3.6 kg, Lidköping, Farms, Lidköping, Sweden) was used. Six rabbits were fed with a diet, “rabbit chow enriched with 0.25% cholesterol” (Analycen AB, Lidköping, Sweden), known to cause atherosclerosis, for 2 weeks before the entire aorta was deendothelialized with repeated passage of a 3F embolectomy catheter. The cholesterol-enriched diet was continued for four more weeks after the deendothelialization injury. Angioplasty was performed in a 3 cm long segment of the infrarenal aorta (30 × 3 mm balloon, VIVA angioplasty catheter, Boston Scientific), and after totally 6 weeks the cholesterol-enriched diet was stopped, and the animals were fed with regular diet. The location of the expanded balloon in the aorta was documented with X-ray angiograms and was evaluated in relation to the lumbar spine and the aortic bifurcation (Figures 1(a) and 1(b)). The initial MRI was performed 2-3 days after PTA, and four weeks later all animals were subjected to a second MRI examination. Three of the rabbits were followed for 6 more weeks (i.e., 12 weeks after deendothelialization and 10 weeks after PTA). At that time an additional MRI analysis was performed and the rabbits were sacrificed by an overdose as described below. 

The first half (*n* = 3) of study group was euthanatized after 6 weeks after deendothelialization, the vasculature were perfused at 120 mm Hg with ice-chilled phosphate-buffered saline followed by a fixative (4% paraformaldehyde in phosphate buffer) and the aorta processed further for histological analysis. The second half (*n* = 3) of the study group were continued on the diet for 6 more weeks (i.e., 10 weeks after angioplasty). All animal interventions were performed at the Department of Diagnostic Radiology Animal Laboratory, Uppsala, Sweden and the study protocol was approved by the local Ethics Committee for animal experiments. 

### 2.2. Anesthesia and Medications

For deendothelialization and angioplasty procedures, the animals were anesthetized with 0.33 mL/kg subcutaneous Hypnorm (0.315 mg/mL fentanyl and 10 mg/mL fluanosine, Janssen Pharmaceutica) and 0.33 mL/kg intramuscular Dormicum (midazolam, 5 mg/mL, Roche), and the anesthesia was maintained with intermittent intravenous bolus doses. Preoperative metronidazole (125 mg, Zinacef, Glaxo Wellcome) was administered and the animals were heparinized (1000 IU) before catheterization. All MRI procedures were performed under slight fluanisone/fentanyl and midazolam sedation. At the study end-point, full surgical anesthesia was induced as above and the animals were euthanatized with an overdose of fluanisone/fentanyl and midazolam-anesthesia. 

### 2.3. MRI

The animals were examined with a Philips Gyroscan ACS NT-II imager with field strength of 1.5 T using a knee coil. They were anesthetized with a mixture fluanisone/fentanyl and midazolam sedation of ketamine administered by repeated i.v. injection during the MRI examination. The same MRI technique as described in our previous studies was used, and the examination covered a 10 cm segment of the aorta with its caudal border located at the aortic bifurcation [1, 2]. To define the region of interest, three different survey sequences were used which facilitated correct positioning of the inflow angio sequence (M2DI) and the proton density weighted sequence (PDW). Both of these sequences were used to evaluate the aortic wall. For the comparison between MRI-examinations, a 2 cm section, 6 axial slices, located centrally within the PTA-affected part of the vessel was selected and followed over time with repeated measurements. For each rabbit a 2 cm long segment of the aorta cranial to the PTA-affected section, containing 6 axial slices from each of the imaging series, was selected as control. For each individual animal 6 slices from each region were compared. 

The M2DI sequence showed the flowing blood in the vessel with high signal intensity and the PDW visualised the surroundings with different intermediate signal intensities. The PDW sequence, a turbo spin echo sequence, was applied in two consecutive acquisitions, the second filling the gaps in the first acquisition. 

### 2.4. Image Processing

**Figure 2:**
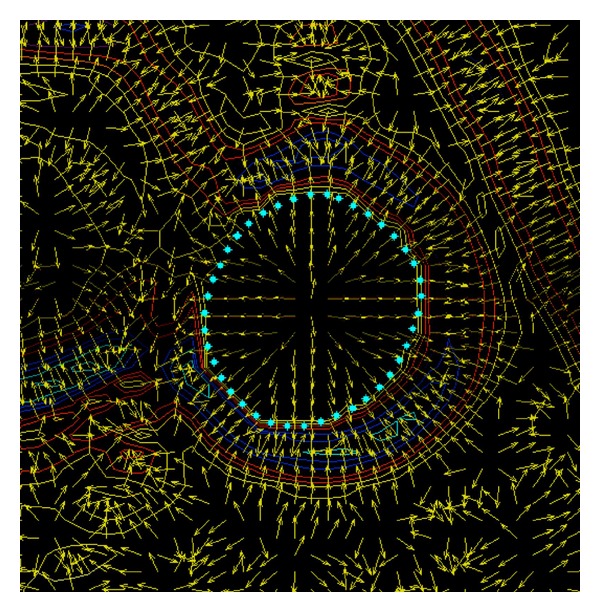
Gradient forces in a PDW image affecting the GVF snake. Curves of different colour indicate different gray-levels in the image, yellow arrows represent the external forces acting on the snake during its motion, and cyan star signs mark the final equilibrium position of the snake.

**Figure 3:**
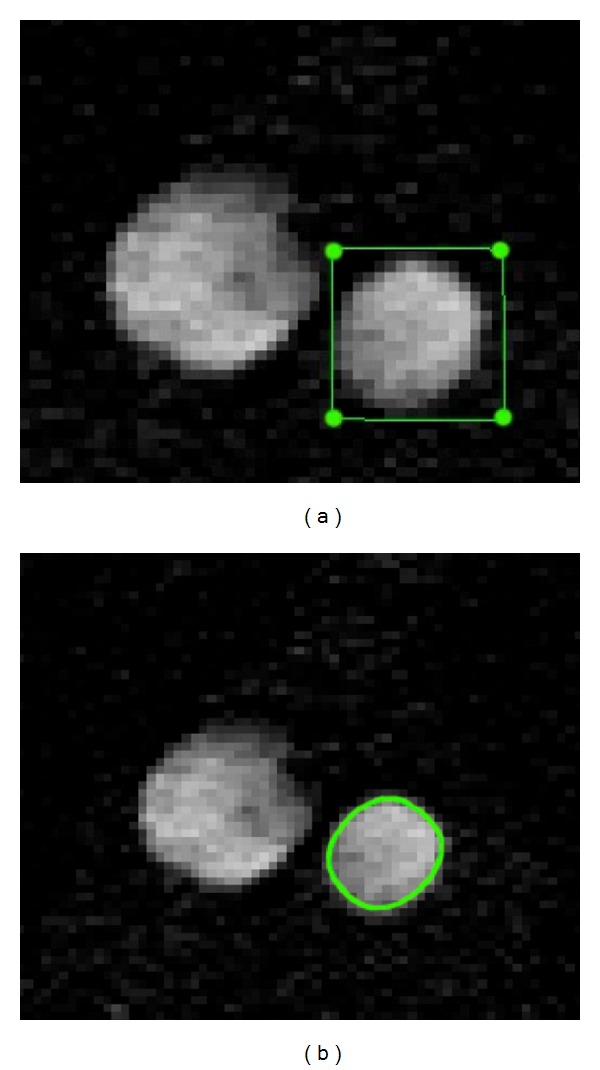
GVF-snakes applied to M2DI sequence. (a) The dots are the starting points applied nearby the aorta. (b) Green line shows the resulting GVF-snake after automatic segmentation.

**Figure 4:**
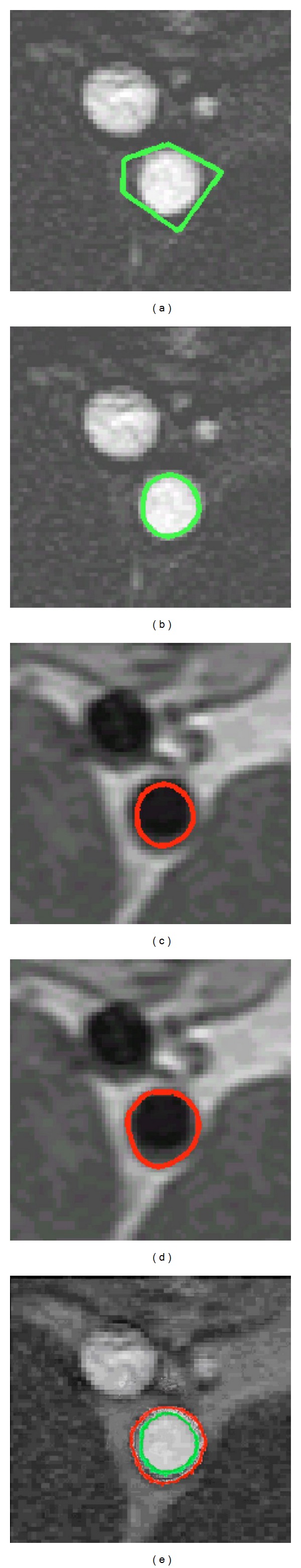
Example of the GVF snake algorithm. (a): A coarse approximation is drawn in the first M2DI slice. (b, c) The final position of the snake in this slice (b), corresponding to the inner boundary of the vessel wall, is transferred to the next M2DI slice and also to the first PDW slice (c). (d, e): The final position of the snake in this image (d) represents the outer border of the vessel wall, and the area between the two snakes (e) is a measure of the wall thickness.

Image processing was accomplished with a Gradient Vector Flow (GVF) snake algorithm [3] (cf.  Figure 2). The objective of the image processing was to identify the inner and outer boundaries of the vessel wall. The inflow angio sequence shows stationary tissue (both vessel wall and surrounding tissue) with low signal and flowing blood in the aorta and the inferior vena cava, with high signal intensity (Figure 3). The snake is a dynamic contour that is attracted to regions in the image, which are rich in edge information [4]. When the snake is applied to the MRI images, it will seek the borders between tissues that differ in signal, such as the inner and outer border of the vessel wall, in the inflow angioimages. In the PDW images the surroundings of the lumen are presented by various intermediate signal intensities. The snake will seek the areas in the image which have most edge information, thereby identifying the outer border of the vessel wall, since this seems to be the area where the major edge information is located (Figures 4(a)–4(e)). A complete program for dealing with and segmenting the images was implemented in Matlab and run on a Sun Ultra 30 workstation. 

### 2.5. Histology

After the vasculature was perfused with the fixative, the length of the infrarenal aorta was measured *in situ *in each animal from the bifurcation to the left renal artery. Thereafter this vessel segment was divided every 10 mm beginning from the distal end. Specimens were then embedded in paraffin, sectioned in 4-5 *μ*m slices, and stained with hematoxylin and eosin. In the histopathology images wall intima-media area and thickness was measured by delineating the contours. Histopathology measurement corresponding to a single MRI-slice were combined by calculating the mean value. 

### 2.6. Statistical Methods

The development of MRI-measured wall area and wall thickness was evaluated with analysis of variance, allowing for variation between individuals as well as between the measured slices within the PTA-affected, 6 MRI-slices, and proximal segments, 6 MRI-slices, and change over time (repeated measures). The limit of significance was set at *P* = 0.05. Wall area measured with MRI was correlated to wall intima-media area measured with histopathology by calculating a correlation coefficient (Pearson *r*), based on all the MRI slices with corresponding histopathology.

## 3. Results 

**Figure 5:**
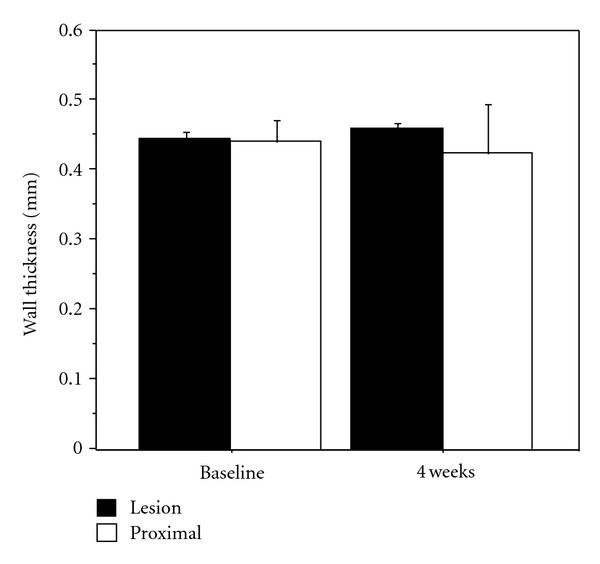
MRI-snakes-measured arterial wall thickness (mm) in lesion and proximal segment at baseline and 4 weeks. Mean values calculated from 6 slices for each of the 6 animals, 36 slices from PTA-treated region, and 36 slices from non-affected region.

**Figure 6:**
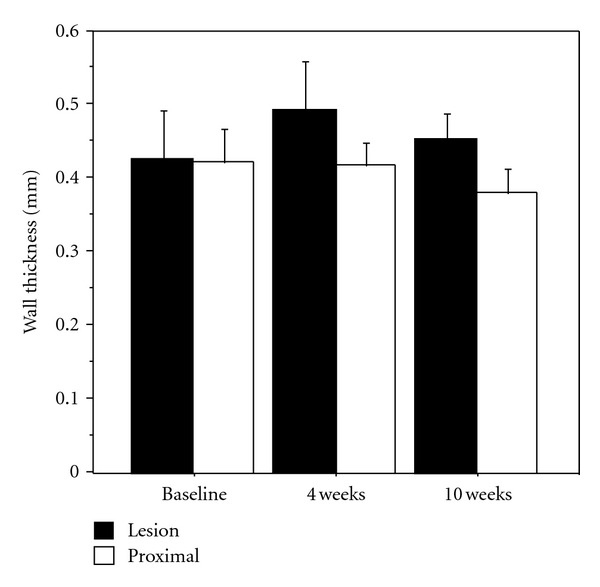
MRI-snakes-measured arterial wall thickness (mm) in lesion and proximal segment at baseline, 4 and 10 weeks, mean values for 3 animals, 18 slices from PTA-treated region, and 18 slices from proximal region.

**Figure 7:**
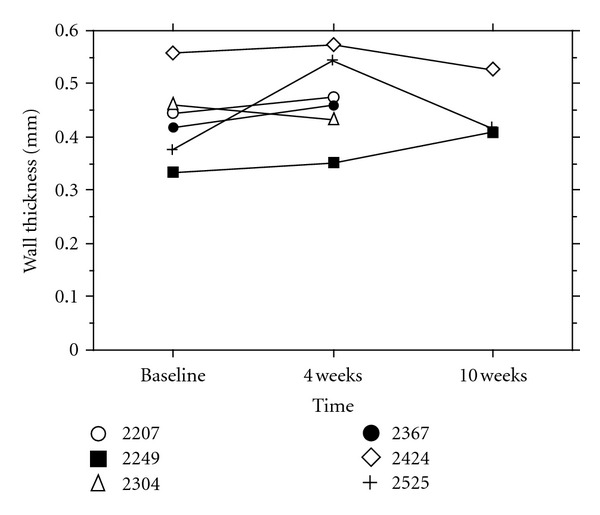
MRI-snakes-measured arterial wall thickness in lesion at various points in time for each individual animal, 6 slices from each region.

**Figure 8:**
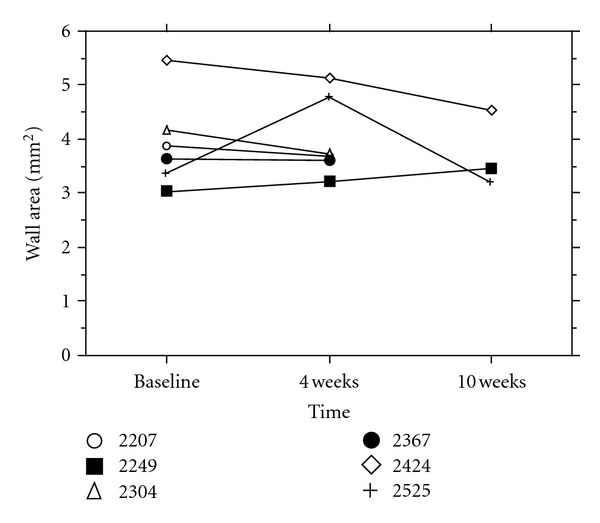
MRI-snakes-measured arterial wall area in lesion at various points in time for each individual animal, 6 slices from each region.

At baseline, there was no significant difference in wall thickness between the lesion and the proximal piece. Wall thickness within the lesion, measured with MRI 4 weeks after PTA (in all 6 animals, 36 MRI-slices from each region), was significantly larger compared to the measurement from the lesion at baseline and to proximal vessel wall thickness at simultaneous examination (Figure 5). Wall thickness measured at 10 weeks (in 3 animals, 18 slices from each region) was not significantly different from the measurement at 4 weeks but significantly larger than thickness in the segment proximal to the lesion, 36 and 18 slices, respectively (Figures 6 and 7). Wall thickness measured proximal to the lesion did not change significantly between different points in time (Figure 6). The wall area in the lesion did not change significantly from baseline to 4 weeks (*n* = 6), but decreased significantly from 4 to 10 weeks (*n* = 3) (Figure 8). The correlation between histological intima-media thickness and the last MRI-snakes-measured wall area was *r* = 0.89 (*P* < 0.0001). 

## 4. Discussion 

When technically possible, PTA is often chosen as the first intervention for a patient with symptomatic arteriosclerosis [5]. Restenosis after PTA is a well-known complication for which several different types of treatments are available for the patients [6–9]. The onset of atherosclerosis can be silent and the early stages of restenosis can also elapse silently. The purpose of our study was to find out whether it is possible to follow vessel wall changes post-PTA over time, during the first 10 weeks after PTA, in an animal model. The complexity of histopathology in atherosclerosis is known and has been studied by several groups [1, 10–15]. The invasive *in vivo* methods available for animal models include angiography, intravascular MRI and ultrasound (US) [16–20]. Angiography is regarded as the gold standard when hemodynamic conditions are evaluated [21, 22]. Although angiography can diagnose calcifications in the vessel wall information about other compounds in the vessel wall is very limited. Electron-Beam Computed Tomography and multi-slice helical computerized tomography [23–25] are methods that could be used noninvasively for measuring the amount of calcifications in the vessel wall, but these methods also do not produce information about vessel wall thickness and/or area. Risk factors associated with the method such as the X-ray exposure and the use of iodinated contrast media must also be considered. 

Intravascular US and MRI are promising modalities when assessing the amount of different components in the vessel wall. The main problem for US is anatomical orientation [26–30]. The location of probe position has to be described in relation to the surroundings or distance from closest situated vessel branches. Also determining the vessel wall measurement in relation to different layers in the wall can be difficult, since the ultrasound is completely reflected if calcifications are present in the wall. Artefacts from calcifications could create loss of information from adjacent tissues and consequently a loss of information about the wall composition. US is also operator-dependent to a considerable degree [26, 31]. Percutaneous ultrasound with Doppler is by far the method most used in clinical practice when hemodynamic conditions are evaluated [27, 28, 32]. Percutaneous ultrasound can be applied in animal models [33], but because of the inter-observer variability it is not commonly used in animal studies. Intravascular MRI can be used for measuring and diagnosing vessel wall morphology [18, 19]. Although the modality is less operator dependent, its invasive approach limits its use. 

During the last decades several groups have presented interesting results with MRI used in a non-invasive way to examine vessel wall morphology [34–41]. In our study we decided to use a non-invasive quantitative method applied to an animal model to assess vessel wall changes at different points in time. The PTA-affected site was located in the distal part of the abdominal aorta and a 2 cm long section of the abdominal aorta cranial to the PTA location was considered as a control region. The double injury, deendothelialization during cholesterol-enriched diet followed by PTA, initiates different vessel wall changes at different locations [42, 43]. In the control region, cranial to the lesion, a major change in the intimal layer could be expected, since the deendotheliazation causes neointima development. The media layer should not be affected. In the PTA-affected region the vessel wall reaction at 4 weeks control could be a sign of intimal thickening and instigation of change in the medial layer. The reaction in the intimal layer would decrease through following period of time. We recorded the highest values for both thickness and area measurements at 4 weeks. The thickness and area were somewhat smaller at 10 weeks than both the initial and the second measurement. A potential source of error in this study is the location of the PTA-balloon. By using X-ray films from the PTA session and the images from the MRI survey sequences, it was possible to correlate the position of the balloon to the vertebral bodies in the lumbar spine, Figures 1(a) and 1(b). The balloon, 30 × 3 mm, would affect the vessel wall in at least 10 MRI slices. By selecting six of the slices located in the middle of the part of the aorta corresponding to the balloon location, we assume that the studied slices represent PTA-affected vessel and may be compared with six slices surely proximal to the lesion. It is not clear to what extent motion artifacts from respiration affect the results and whether there are ways of reducing this. 

A limitation of our method is that it cannot distinguish between the intima and media in the arterial wall, which can be done by ultrasound [27, 29]. However, when only information about the effect on total wall thickness is required, the proposed method appears to be an attractive alternative to histopathological studies, as the same individual may be followed over time, resulting in better statistical power for a given sample size and requiring smaller number of subjects. 

In conclusion, the proposed *in vivo* method may detect the amount of initial vessel wall changes post-PTA. In future studies, it would be interesting to follow a larger number of animals, thus a higher total of measurements, for a longer period of time and to perform corresponding histopathology analyses. The ultimate goal is to develop an *in vivo* method that could be used clinically to measure wall thickness/area and also provide morphological information of the vessel wall.
